# Suppressors of cytokine signaling in tuberculosis

**DOI:** 10.1371/journal.pone.0176377

**Published:** 2017-04-21

**Authors:** Shih-Wei Lee, Chi-Wei Liu, Jia-Ying Hu, Li-Mei Chiang, Chih-Pin Chuu, Lawrence Shih-Hsin Wu, Yung-Hsi Kao

**Affiliations:** 1 Department of Life Sciences, National Central University, Taoyuan, Taiwan; 2 Taoyuan General Hospital, Ministry of Health and Welfare, Taoyuan, Taiwan; 3 Institue of Cellular and System Medicine, National Health Research Institutes, Miaoli, Taiwan; 4 Institute of Medical Sciences, Tzu Chi University, Hualien, Taiwan; South Texas Veterans Health Care System, UNITED STATES

## Abstract

Tuberculosis (TB), a global disease mainly infected by *Mycobacterium tuberculosis*, remains leading public health problem worldwide. Suppressors of cytokine signaling (SOCSs) play important roles in the protection against microbial infection. However, the relationship between members of the SOCS family and tuberculosis infection remains unclear. Using peripheral blood mononuclear cells, we investigated the mRNA expression profiles of SOCS subfamilies among active TB, latent tuberculosis infection (LTBI), and healthy individuals. Our results showed that active tuberculosis subjects had higher levels of SOCS-3 mRNA, lower expressions of SOCS-2, -4, -5, -6, -7, and cytokine-inducible SH2-containing protein-1 (CIS-1) mRNAs, but not SOCS-1 mRNA than healthy and LTBI subjects. In men, LTBI patients had lower SOCS-3 than healthy subjects, and active TB patients had lower levels of SOCS-4, -5, and CIS-1 mRNAs but higher levels of SOCS-3 mRNA than healthy subjects. In women, LTBI patients had lower SOCS-3 mRNA level than healthy subjects, and active TB patients had lower CIS-1 mRNA level than healthy subjects. In non-aged adults (< 65 years old), TB patients had higher SOCS-3 mRNA and lower levels of SOCS-2, -4, -5, -6, -7, and CIS-1 mRNAs; whereas, aged TB patients (≥ 65 years old) had lower levels of SOCS-5 and CIS-1 mRNAs. These data suggest that particular SOCS members and their correlative relationships allow discrimination of active TB from healthy and LTBI subjects.

## Introduction

Tuberculosis (TB) is a global disease infected by various strains of mycobacteria, especially *Mycobacterium tuberculosis* (*Mtb*) [1]. One third of the world’s population is latently infected with *Mtb*; about 90% of infected individuals are asymptomatic and up to 10% of infected individuals progress to active TB, which is characterized by a chronic cough with blood-containing sputum, fever, night sweats, and weight loss [1, 2]. Although the tuberculin skin test is the most common diagnostic test for the detection of latent tuberculosis infection (LTBI), it has a tendency to produce false-positive results due to the administration of BCG vaccine or other viral infections [1, 3]. Thus, a semi-quantitative polymerase chain reaction method was used to detect changes in the expression of particular genes [4] which allow discrimination of active TB from LTBI subjects, as well as helping distinguish healthy individuals from active TB and LTBI subjects.

Suppressor of cytokine signaling (SOCS) is a src homolog 2 (SH2) domain-containing polypeptide that was found to inhibit cytokine signaling through negative feedback [5]. Since its discovery, it was found to possess numerous actions and was reported to constitute the SOCS protein family with other SH2 domain-containing and SOCS box-containing proteins, SOCS-1, -2, -3, -4, -5, -6, -7, and cytokine-inducible SH2-containing protein (CIS)-1. For examples, SOCSs modulate immunity and inflammation and provide negative feedback to suppress cytokine [i.e., interleukin (IL)-1, -6 and interferon gamma] and hormone signaling [5]. Recent studies have indicated that SOCS-1 and -3 were higher in active TB patients than healthy subjects [6, 7]; the expression of SOCS-1 gene is associated with caseous necrosis in granulomas from patients with TB lymphadenitis [7]. Despite of this attention, no studies have demonstrated whether other SOCS family members closely associate with TB and whether their mRNA expression profiles differ from healthy subjects. No reports have found whether any of SOCS expression is different between LTBI and active TB patients living in Taiwan.

In this study, we used peripheral blood mononuclear cells (PBMCs) to investigate the differences in the expression of SOCS genes among healthy individuals, LTBI subjects, and active TB patients in Taiwan. We showed that expressions of SOCS-2, -3, -4, -5, and CIS-1 mRNAs, but not SOCS-1, -6, or -7 mRNAs, were significantly different between healthy individuals and TB subjects, or between active TB and LTBI subjects. We also found that the mRNA expression profiles of particular members of the SOCS family varied with the gender and age.

## Methods

### Clinical sample collection

All procedures were reviewed and approved by the Institutional Review Board of Taoyuan General Hospital, Ministry of Health and Welfare, Taoyuan, Taiwan. Written informed consents were obtained from all participants. Eligibility for entry into the study was based on clinical signs and symptoms of *Mtb* infection. LTBI subjects were recruited from close contact with active TB patients, with positive T-SPOT TB test and negative chest radiograph, but without clinical evidence of active TB. Healthy controls (HC) were individuals who had not been in close contact with TB or LTBI patients and showed no clinical signs of TB or LTBI. Individuals with allergic diseases, diabetes, cancer, immunocompromised conditions, and coinfections with any types of infectious diseases were excluded. In total, 35 healthy individuals (16 men and 19 women), 29 subjects with LTBI (12 men and 17 women), and 42 subjects with active TB (30 men and 12 women) were enrolled in this study. The means ± SD of age in the healthy, LTBI and active TB subjects were 50.3 ± 17.7, 28.8 ± 9.8, and 56.8 ± 21.0, respectively. Because LTBI patients mostly belonged to contacts of TB patients, in which some of LTBI and TB participants were collected from the same family, the LTBI patients would be younger than active TB patients.

### RNA isolation

According to the methods of Wu et al [8], we isolated RNA from PBMCs. RNA quality was determined by an optical density (OD) 260/280 ratio ≥ 1.8 and OD 260/230 ratio ≥ 1.5 on a spectrophotometer and by the intensity of the 18S and 28S rRNA bands on a 1% formaldehyde-agarose gel. RNA was subjected to real-time PCR analysis.

### Real-time polymerase chain reaction (real-time PCR) for SOCS mRNA

According to the methods of Chang et al [9], we used real-time PCR to determine the levels of SOCS-1, -2, -3, -4, -5, -6, -7, CIS-1, and GAPDH mRNAs. cDNA was synthesized from equal amounts (5 μg) of RNA using 100 units of M-MLV reverse transcriptase (Invitrogen) in the presence of 40 units of RNase inhibitor (Invitrogen) and the adapter primer was 5’-GGCCACGCGTCGACTAGTAC (T)19-3’. According to the manufacturer’s two-step cycling protocols, the real-time PCR analysis was performed twice in duplicate by use of the power SYBR green PCR master mix (Kapa Biosystems, Boston, MA) and ABI 7300 Sequence Detection System (Applied Biosystems, Foster City, CA) under the following conditions: an initial denaturing cycle at 95°C for 5 min, followed by 40 cycles of amplification consisting of denaturation at 95°C for 3 s and annealing/extension/data acquisition at 60°C for 30 s. The forward and reverse primers are showed in Table 1. Normalization involved GAPDH mRNA levels as controls in parallel reactions. The relative expression ratio of SOCS transcripts to GAPDH transcript was calculated as described by Chang et al [9], and then expressed as the percent of the control.

**Table 1:** Primers for the detection of suppressor of cytokine signaling (SOCS), cytokine-inducible SH2-containing protein-1 (CIS-1), and GAPDH gene expression by real-time PCR.

Genes	Sequence of forward primers (FP) and reverse primers (RP)	Accession number (sizes of PCR product)
*SOCS-1*	FP: 5’-CTGGGATGCCGTGTTATTTT-3’	NM_003645.1
	RP: 5’-TAGGAGGTGCGAGTTCAGGT -3’	(245 bp)
*SOCS-2*	FP: 5’-GCTCGGTCAGACAGGATGGT -3’	NM_003877.3
	RP: 5’-TTGGCTTCATTAACAGTCATACTTCC -3’	(51 bp)
*SOCS-3*	FP: 5’-GGGGAGTACCACCTGAGTCT-3’	NM_003955.3
	RP: 5’-CGAAGTGTCCCCTGTTTGGA-3’	(128 bp)
*SOCS-4*	FP: 5-CACTCTTCAGGGCTTCCGTC-3’	NM_080867.2
	RP: 5’-AGGCTAAATCTGATCGAGGTGG -3’	(91 bp)
*SOCS-5*	FP: 5’-ATCTGGAGACAGCCATACCCA -3’	NM_014011.4
	RP: 5’-CAAATCAGGCACGAGGCAGT -3’	(91 bp)
*SOCS-6*	FP: 5’-CGCTAGCCAGTGACTTTGGA -3’	NM_004232.3
	RP: 5’-TCTGTTTTGCAGAAAGCCGC -3’	(165 bp)
*SOCS-7*	FP: 5’-CCTTCAGCCTGTGGTGTCAT -3’	NM_014598.2
	RP: 5’-TCGGGACACTGGATAGAGCA-3’	(170 bp)
*CIS-1*	FP: 5’- GGTACCCCAGCCCAGACAGAGA-3’	NM_013324.5
	RP:5’-GGAACCCCAATACCAGCCAGAT-3’	(114 bp)
*GAPDH*	FP: 5’-GGAGCCAAAAGGGTCATCATCTC-3’	NM_001256799.1
	RP: 5’-GAGGGGCCATCCACAGTCTTCT-3’	(232 bp)

### Statistical analysis

Data are expressed as the box and whiskers plots with 10-90^th^ percentiles, the outliers, and median and mean values of each group shown by a horizontal line. One-way ANOVA followed by the Student-Newman-Keuls multiple-range test were used to examine differences among multiple groups. Student *t*-test was used to examine differences in sex and age. Differences were considered significant at *P* < 0.05. Pearson correlation analysis was used to examine correlations between different SOCS molecules. Statistics were performed using SigmaPlot (Jandel Scientific, Palo Alto, CA) and SPSS software (SPSS Inc., Chicago, IL).

## Results and discussion

**Fig 1:**
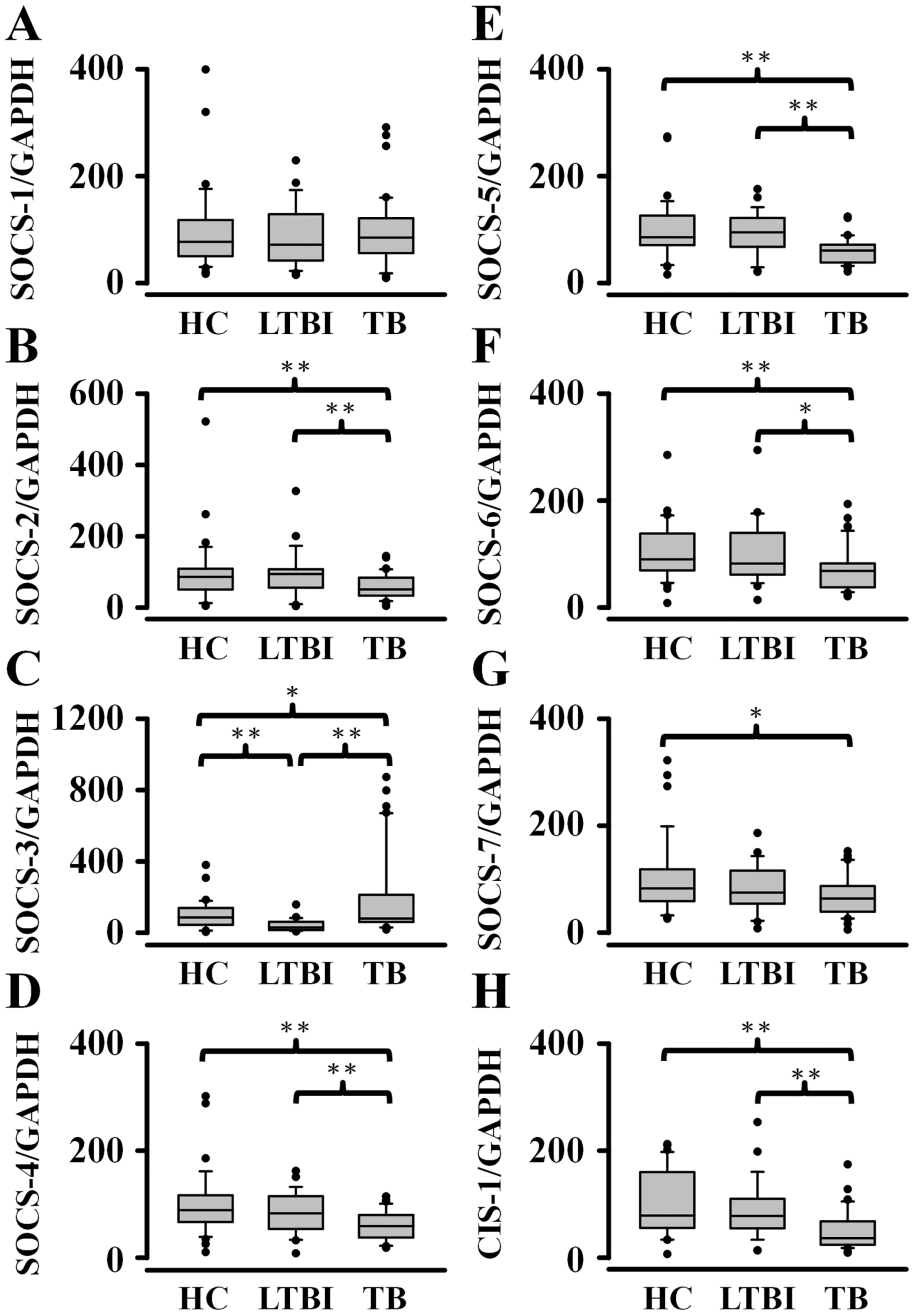
Expression of suppressor of cytokine signaling (SOCS) genes was significantly different among non-infected healthy control (HC) individuals (n = 35), latent tuberculosis infection (LTBI) subjects (n = 29), and active tuberculosis (TB) patients (n = 42) by real-time PCR analysis. Data are expressed as box and whiskers plots with 10-90^th^ percentiles, the outliers, and median (solid line) values of each group shown by a horizontal line. *P<0.05, **P<0.01.

The mRNA expression profiles of SOCS genes in the healthy, LTBI and active TB subjects were presented in the Fig 1. There were no significant differences in the SOCS-1 gene expression among healthy, latently infected, and TB subjects (Fig 1). Expression of no SOCS family members except SOCS-3 (100% vs. 41%) was different between healthy and LTBI subjects. However, the levels of SOCS-2, -3, -4, -5, -6, -7, and CIS-1 mRNAs were significantly different between healthy and TB subjects. TB subjects had lower levels of SOCS-2, -4, -5, -6, -7, and CIS-1 mRNAs but higher levels of SOCS-3 mRNA than healthy individuals. In addition, significant differences were found in the levels of SOCS-2 (60% vs. 92%, *p*<0.01), SOCS-3 (187% vs. 41%, *p*<0.01), SOCS-4 (60% vs. 83%, *p*<0.01), SOCS-5 (59% vs. 94%, *p*<0.01), SOCS-6 (71% vs. 99%, *p*<0.05), and CIS-1 (48% vs. 91%, *p*<0.01) when we compared active TB with LTBI subjects.

**Fig 2:**
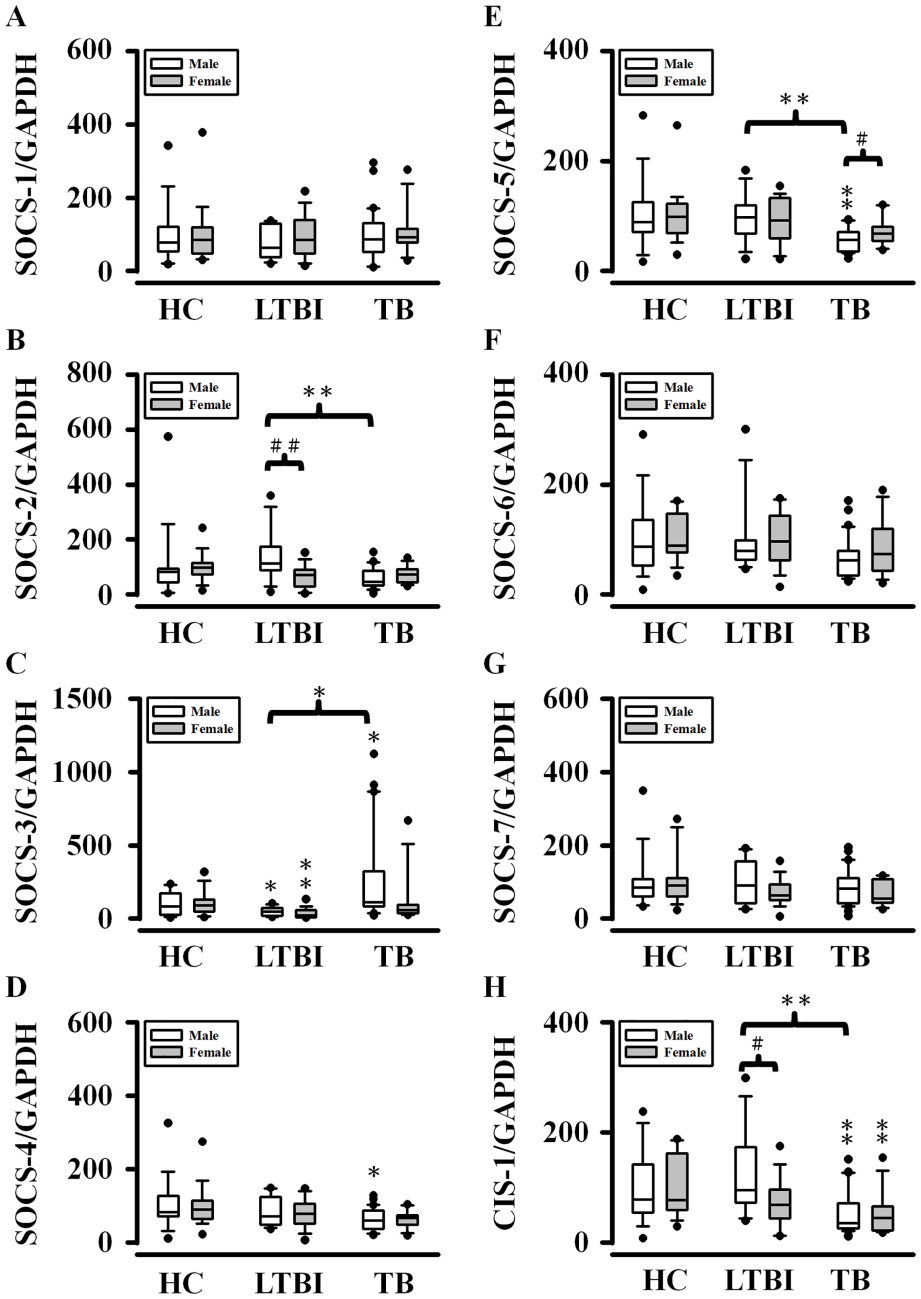
Differences in the gene expression of the SOCS family among non-infected healthy control (HC) subjects (16 men and 19 women), latent tuberculosis infection (LTBI; 12 men and 17 women), and active TB patients (30 men and 12 women) were dependent on the gender. Data are expressed as box and whiskers plots with 10-90^th^ percentiles, the outliers, and median (solid line) values of each group shown by a horizontal line. *P < 0.05 and **P < 0.01, vs HC in a given sex; ^#^*P* < 0.05, men vs. women in a given healthy, LTBI, or active TB subject.

According to the report for Taiwan tuberculosis in 2013 [10], the incidence of TB new cases was higher in men than women [10]. It is unknown whether SOCS gene expression among HC, LTBI and active TB subjects varies with the gender. Accordingly, we separated men from women to examine the possibility for the differences of SOCS gene expression among the three groups in each sex (Fig 2). In men, we found that the levels of SOCS-1, -6, and -7 mRNAs showed no significant differences among HC, LTBI, and active TB subjects. However, LTBI subjects had lower SOCS-3 than healthy and TB subjects, and revealed higher SOCS-2, -5, and CIS-1 than TB subjects. TB subjects tended to have lower levels of SOCS-2, -4, -5, and CIS-1 mRNAs but higher levels of SOCS-3 mRNA than healthy subjects. In women, none of SOCS-1, -2, -4, -5, -6, and -7 mRNAs showed significant differences among healthy, LTBI, and active TB subjects. However, LTBI patients had lower SOCS-3 mRNA level than healthy subjects, and active TB patients had lower CIS-1 mRNA level than healthy subjects.

Next, we evaluated whether SOCS expression in a given group of healthy, LTBI, or active TB subjects was different between men and women. In healthy subjects, there were no significant differences of any SOCS gene expression between men and women. However, in LTBI patients, men had higher levels of SOCS-2 and CIS-1 mRNAs than women. In active TB subjects, men tended to have lower SOCS-5 mRNA level and higher SOCS-3 mRNA level than women.

Our study supports that the mRNA expression profiles of SOCS subfamilies in active TB subjects vary with the gender. Changes in the SOCS-2, -3, and -5 mRNA levels appeared to be beneficially used for better discrimination of active TB from healthy and LTBI subjects in males and females. A few studies indicated that sex hormones (e.g., androgen and estrogen) can regulate SOCS gene expression in prostate [11] and breast [12] cancer cells. Although testosterone was demonstrated as a TB susceptibility factor in mice system [13], TB patients showed lower levels of testosterone and higher levels of IL-6, IFN-γ, and TGF-β [14]. Further studies are needed to determine whether the association of sex hormones with cytokines explains the gender-dependent differences of SOCS expression between healthy and TB subjects.

**Fig 3:**
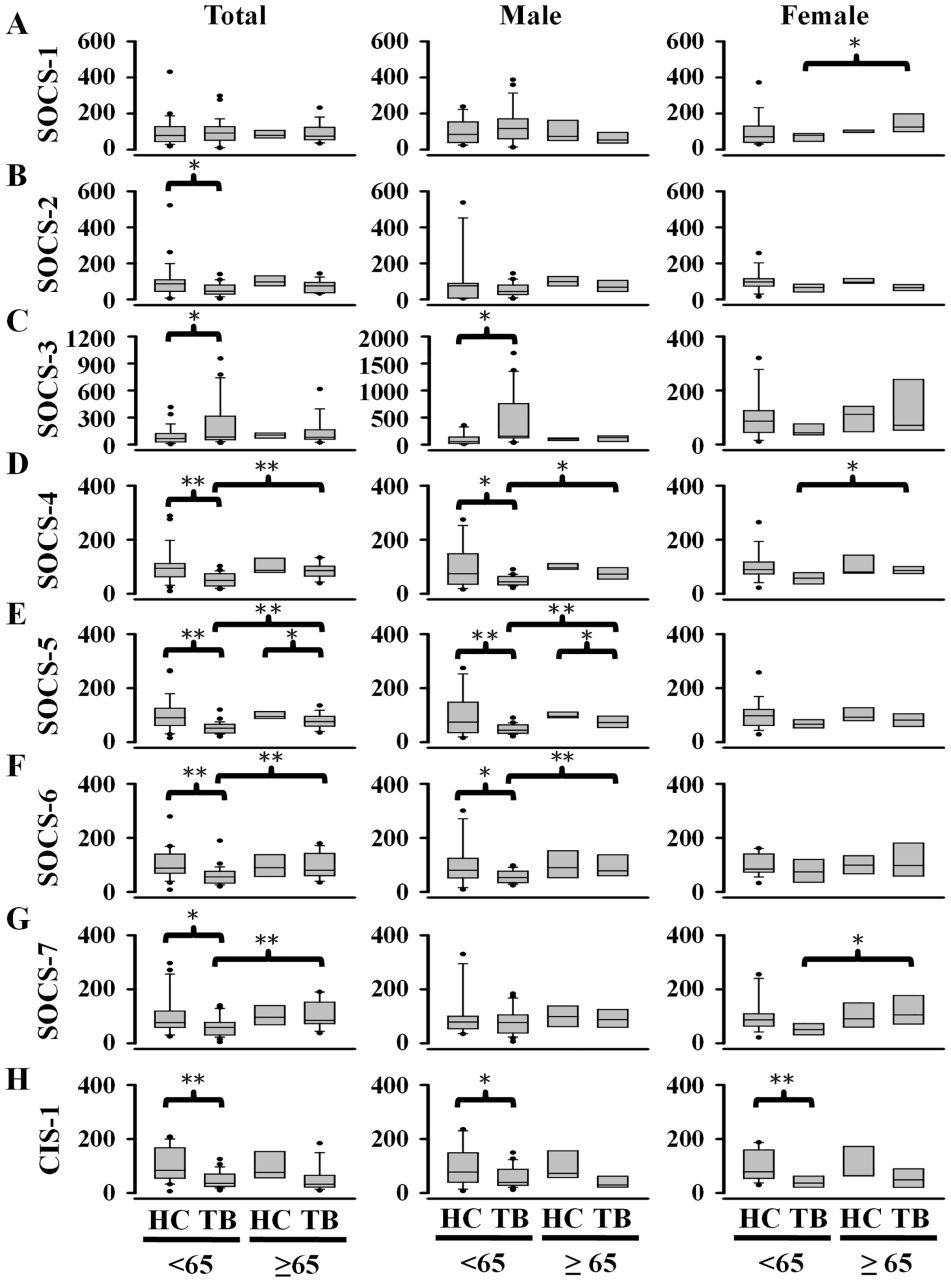
Differences in the gene expression of SOCS family between non-infected healthy control (HC) subjects (non-aged, 11 men and 16 women; aged, 5 men and 3 women) and active tuberculosis (TB) patients (non-aged, 22 men and 6 women; aged, 8 men and 6 women) were dependent on the age when it was cut off at 65 years old. Data are expressed as box and whiskers plots with 10-90^th^ percentiles, the outliers, and median (solid line) values of each group shown by a horizontal line. *P<0.05, HC *vs*. TB in a given age group, or 65 below *vs*. 65 over years old of the active TB patients.

**Fig 4:**
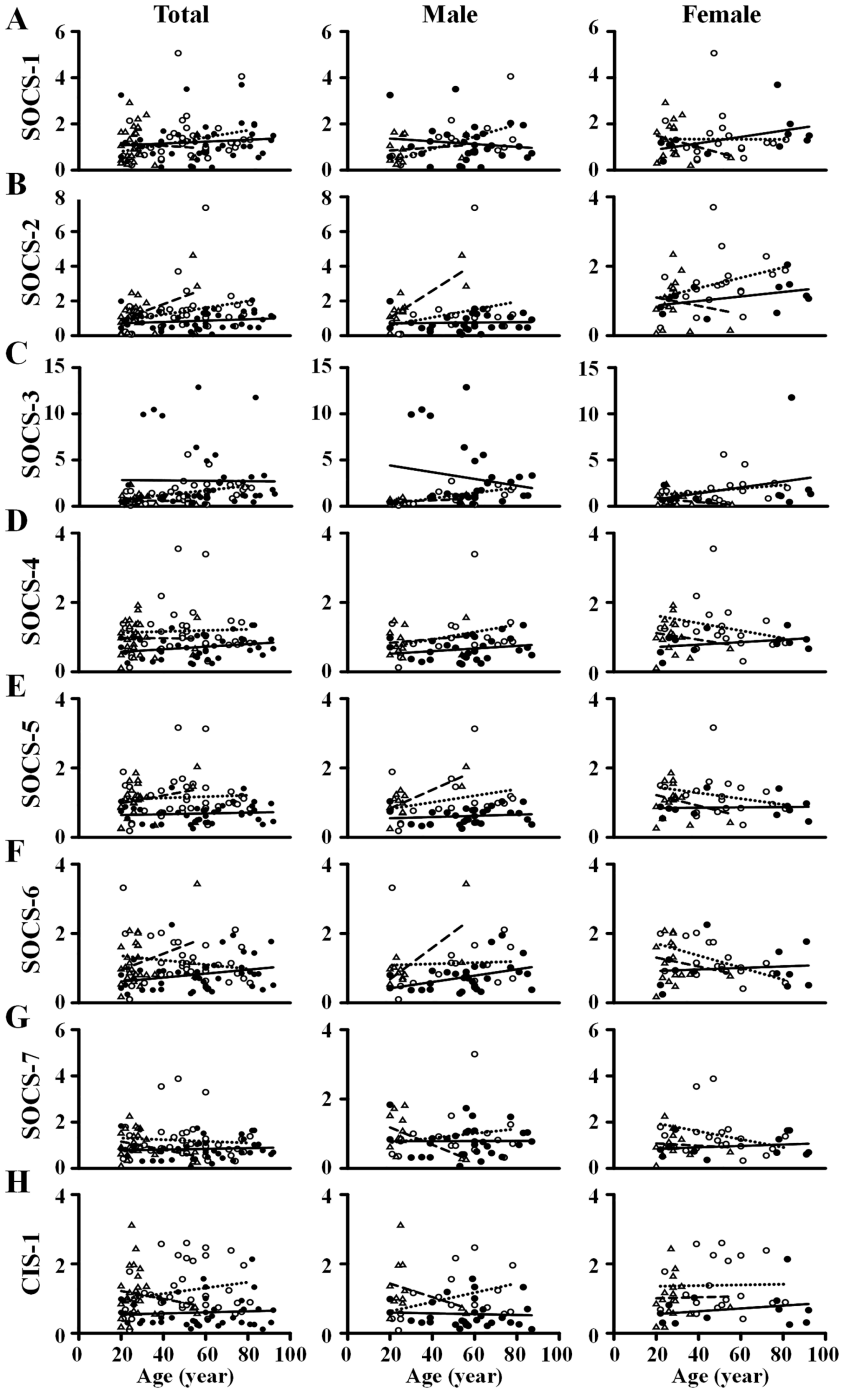
The mRNA expression profiles of suppressor of cytokine signaling (SOCS) family in healthy control (HC) subjects (n = 35; dotted line and open circle), latent tuberculosis infection (LTBI; n = 29; medium-medium line and open triangle) and active TB (n = 42; solid line and solid circle) patients.

According to the report for Taiwan tuberculosis in 2013 [10], the incidence of new TB cases was more than one-half (~52%) in aged subjects (≥ 65 years old) to that of non-aged subjects (< 65 years old). Accordingly, we separated non-aged subjects from aged subjects to examine the possibility for the differences of SOCS gene expression between the two groups (Fig 3). Because the LTBI patients were mostly aged below 65 years old, they were excluded for the comparison. In non-aged subjects, active TB patients rather than healthy individuals had lower levels of SOCS-2, -4, -5, -6, -7, and CIS-1 mRNAs, higher SOCS-3 mRNA levels, and no effects on SOCS-1 mRNA. In men but not in women, active TB patients had significantly higher SOCS-3 mRNA expression than healthy individuals. In aged subjects, active TB patients tended to reveal lower levels of SOCS-5 and CIS-1 mRNAs than healthy participants. Notably, none of the eight SOCS subfamilies had significant differences of their mRNA expression between aged and non-aged healthy subjects. No significant differences were found in levels of SOCS-1, -2, -3, and CIS-1 mRNA between aged and non-aged TB patients. However, SOCS-4, -5, -6, and -7 mRNA levels were significantly different between non-aged and aged TB patients. Taken together, the mRNA expression profiles of SOCS genes during different ages and gender in the healthy, LTBI and active TB subjects were presented in the Fig 4. These observations suggest that the mRNA expression profiles of particular SOCS subfamilies vary with the age in TB patients. SOCS-4, -5, -6, and -7 appear to allow us for discrimination of aged TB patients from non-aged TB patients (Fig 3). A previous study [15] indicated that some genes (e.g., CDO1, HLA-B, CIS-1, IL-17) changed their expressions with age in the human kidney in association with kidney degeneration [15]. Increased levels of the immune-related genes, such as HLA-B, CIS-1, and IL-17, suggests that immune surveillance or inflammation increases with age. It is worthwhile to explore whether the age-dependent differences of these immune-related genes are associated with the distinct expression profiles of SOCS subfamilies between aged and non-aged TB patients.

Correlations between different SOCS molecules in a given group of subjects were summarized in Table 2. In healthy subjects, there were significant positive correlations of 1) SOCS-2 with SOCS-4, -5, -7 and CIS-1, 2) SOCS-4 with SOCS-1, -5, -6, -7, and CIS-1, 3) SOCS-5 with SOCS-1, 6, -7, and CIS-1, and 4) SOCS-7 with SOCS-1 and CIS-1, respectively. In LTBI subjects, there were positive correlations of 1) SOCS-1 with SOCS-3, -4, and -7, 2) SOCS-2 with SOCS-5, 3) SOCS-4 with SOCS-5, -6 and -7, and 4) SOCS-5 with SOCS-6, respectively. Unlike healthy subjects, active TB subjects revealed negative correlations of SOCS-3 with SOCS-4, -5, and CIS-1. There were positive correlations of 1) SOCS-1 with SOCS-2, -4, and -7, 2) SOCS-2 with SOCS-2, -4, -5, -7, and CIS-1, 3) SOCS-4 with SOCS-5, -6, -7, and CIS-1, 4) SOCS-5 with SOCS-6 and SOCS-7, and 5) SOCS-7 and CIS-1, respectively. Thus, correlations between different SOCS molecules may help discriminate active TB from healthy and LTBI subjects.

**Table 2:** Pearson correlations (r) between different SOCS molecules have been observed in a given healthy, LTBI and active TB subjects.

Parameters	Healthy, LTBI, and active TB subjects^a^						
	SOCS-2	SOCS-3	SOCS-4	SOCS-5	SOCS-6	SOCS-7	CIS-1
SOCS-1	0.301 0.048 0.443**	0.235 0.635*** -0.037	0.504** 0.454* 0.358*	0.490** 0.293 0.249	0.171 0.250 0.167	0.381* 0.653*** 0.408**	0.280 -0.164 0.223
SOCS-2		-0.144 0.082 -0.134	0.736*** 0.237 0.549***	0.733*** 0.654*** 0.487***	0.106 0.345 0.117	0.582*** -0.069 0.652***	0.603*** 0.331 0.531***
SOCS-3			-0.191 0.223 -0.378*	-0.136 0.218 -0.337*	-0.166 0.262 -0.166	-0.179 0.321 -0.132	-0.118 -0.041 -0.359*
SOCS-4				0.849*** 0.658*** 0.799***	0.546*** 0.652*** 0.673***	0.830*** 0.446* 0.567***	0.466** 0.127 0.530***
SOCS-5					0.543*** 0.739*** 0.772***	0.622*** 0.099 0.394**	0.629*** 0.367 0.271
SOCS-6						0.315 -0.172 0.058	0.018 -0.019 -0.009
SOCS-7							0.388* 0.077 0.360*

^a^The first, second, and third numbers represent correlation coefficients between two SOCS molecules in healthy, LTBI and active TB subjects, respectively.

*, p < 0.05;

**, p < 0.01;

***, p < 0.001;

-, negative correlation.

We attempted to search the SOCS molecules capable of distinguishing healthy individuals from TB subjects, or LTBI from active TB subjects. The present study not only details expressions of SOCS family members in Taiwanese TB population, but also provides certain understanding of differences of expression of particular SOCS mRNAs among healthy, LTBI and active TB subjects. Higher levels of SOCS-3 mRNA, but not SOCS-1, -6, or -7 mRNAs, in active TB patients than healthy and LTBI subjects were observed. It was previously showed that the SOCS-3-stimulating cytokines IFN-γ, IL-6, and resistin were higher in active TB than healthy subjects [1, 2, 6, 16]. Notably, the SOCS-3 molecule, but not any of other SOCS family members, is the main downstream resistin signaling molecule [17]. Whether this mechanism explains higher expression of SOCS-3 mRNA in active TB than LTBI and healthy subjects was not demonstrated in this study. The observed lower SOCS-3 mRNA levels in LTBI than healthy and active TB subjects may also be attributed to changes in the level of posttranscriptional modification, which is evident by the greater amount of SOCS-3-targeting microRNA hsa-miR-221-3p [8]. Significant variations were also found in the up-regulated or down-regulated expressions of other miR molecules (e.g., has-miR-146a-5p, has-miR-150-5p and has-miR-16-5p) and their predicted target genes (e.g., *FOS* and *CXCL2*) when active TB subjects were compared to healthy and LTBI subjects [8]. Whether these molecules are associated with down-regulated levels of SOCS-2, -4, -5, -6, -7, and CIS-1 mRNAs in active TB subjects is unknown.

In support of this study, we attempted to link the expression of SOCSs to their regulatory downstream molecules that could have been altered by decreased or elevated SOCS expression. For example, the interferon regulatory factor (IRF)-3, IL-1β, IL-4, and IL-10 have been reported by various laboratory studies as the target genes of SOCS-2, -3, -4, and -5, respectively [18–21]. The target genes of CIS-1 were found to include c-fos, whey acidic protein (WAP), and IL-2 receptor beta (IL-2Rβ) [22]. When our data linked to those reported microarray study [23], we indeed found that active TB subjects revealed the lowest expressions of SOCS-2 and SOCS-4 among 3 groups of the clinical subjects in the respective associations with the highest expressions of IRF-3 and IL-4. The SOCS-3 shown in ascending expressive order of this study was LTBI, healthy, and active TB subjects and such an order was strengthened by the findings that the IL-1β were the lowest in LTBI patients and the highest in active TB patients [23]. Among the three clinical subjects, active TB patients possessed the least SOCS-5, CIS-1, c-fos [23], and IL-2Rβ [23] mRNAs, as well as the greatest IL-10 [23] and WAP [23] mRNAs. The close link of particular SOCS family members to their target genes provided somewhat more power to the SOCSs measured in this study and led us to understand more discrimination of active TB from healthy and LTBI subjects.

Our study showed that the levels of SOCS-1 mRNA had no significant differences among healthy, LTBI, and active TB subjects. This finding is consistent with those reported no significant differences of SOCS-1 expression in PBMCs between healthy and severe TB subjects living in London [24] or in Pakistan [6–7, 25]. Notably, our finding is inconsistent with those reported subcellular data that showed greater SOCS-1 expression in T cells of severe TB patients living in Pakistan [25] and that showed greater SOCS-1 expression in neutrophils and monocytes, but not in CD4 or CD8 T cells, of severe TB patients living in London [24]. When PBMCs were isolated from endemic controls and pulmonary TB patients and then stimulated with *M*. *tuberculosis*, respectively, they exhibited greater SOCS-1 expression in TB patients relative to the controls [6]. The *M*. *tuberculosis*-induced SOCS-1 expression in PBMC cells was not observed in patients with extra-pulmonary tuberculosis relative to the controls [6]. Alternative explanations for the discrepancy are that the various types of white blood cells are present in the reported experimental condition of PBMCs and that the distinct types of white blood cells express SOCS-1 at varying levels between healthy and TB subjects in response to the *in vivo* or *in vitro* stimulations of lymphokines and *M*. *tuberculosis* [6–7, 24–25]. Further subcellular analysis will help clarify whether SOCS-1 is differentially expressed in T cells, neutrophils, and monocytes between healthy and TB subjects living in Taiwan.

Differential activation or inhibition of immune responses by SOCS family members have been reported [26–30]. SOCS-1 negatively regulates the differentiation of helper T cells (Th) 1 and 2, but promotes Th17 differentiation [26–27]. SOCS-2 inhibits Th2 differentiation and maintains the stability of the regulatory T cells [28]. Transgenic SOCS-3 expression inhibits Th1 and Th17 differentiation and promotes Th2 development, while SOCS-3 silencing attenuated the Th2 response and promotes Th17 differentiation [26–27]. Transgenic SOCS-5 expression suppresses IL-4-induced Th2 differentiation. SOCS-6 controls T cell receptor-mediated T cell activation, and SOCS-7 knockout mice possess increased mast cell numbers [29]. The CIS-1 transgenic mice develop defects in T-cell and natural killer (NK) cell development [22]. The SOCS family was also reported to regulate the function and development of macrophages, dendritic cells, and microglial cells [30]. Accordingly, our findings that active TB subjects possessed higher SOCS-3 expression and lower SOCS-2, -4, -5, -6, -7, and CIS-1 mRNA levels than healthy subjects support the existence of the different immune responses to cytokine signaling between two groups of subjects and suggest the favorite Th2 development in TB subjects. The notion is consistent with those reported for changes in the cytokine profiles of Th cell subpopulation in tuberculosis [31–32]. We should note that the observed lowest CIS-1 mRNA levels in PBMCs of the Taiwanese TB patients is inconsistent from those reported greater CIS-1 expression in T cells of the Germany TB patients relative to LTBI patients [33]. As PBMCs used in our experiment contain a variety of white blood cells that express varying levels of CIS-1 and its upstream signal elements, we could not exclude the possibility that the presence of the subtypes of T cells and the other types of white blood cells may help explain our observed differential CIS-1 expression between healthy and TB subjects. As CIS-1 promoter polymorphisms, particularly rs414171 and rs809451, could regulate CIS-1 expression in lymphocytes and contribute to the susceptibility to tuberculosis in Han Chinese population [34], this genetic mechanism may also help explain lower CIS-1 expression in Taiwanese TB patients.

Among the subjects screened at our clinic, 106 fulfilled the inclusion and exclusion criteria. There are 35 healthy individuals (16 men and 19 women), 29 LTBI subjects (12 men and 17 women), and 42 active TB subjects (30 men and 12 women) enrolled in this study. Another possible explanation for the exclusion is that some RNA samples from the healthy and LTBI subjects were not collected with a good quality for PCR analysis. This may cause the slight difference in the male: female ratio in healthy, LTBI and TB to be about 46:54, 41:59, and 71:29, respectively. Under the similar male: female ratio, LTBI but not healthy subjects indicated gender-dependent differences of SOCS-2 and CIS-1 mRNA levels. Interestingly, active TB groups with reversed male: female ratio compared to the other groups had no significant differences in both SOCS genes that showed gender-specific differences in LTBI group. Whether the male: female ratio explains the expression difference of particular SOCS molecules in healthy, LTBI and TB subjects is possible, but this requires further demonstrations. Notably, according to the means and the number of subjects, the levels of the statistical power for total subjects, genders, and age ranged from 0.81–1 (except the power was 0.73 when the SOCS-3 was examined with a comparison between healthy and active TB subject), from 0.66–1, and from 0.64–1, respectively. Although the total number of subjects provided sufficient power (>0.8) and the variations were smaller in many of the small sample size grouped subjects, we could not exclude the possibility that the number of subjects in some groups used in this study may be too low to separate them by age and/or gender while keeping sufficient power. Thus, a future study to further increase the number of each grouped subjects will help solidify the gender- and age-dependent differences of the SOCS molecules among healthy, LTBI, and TB subjects.

## Conclusions

Early important reports have extensively indicated many candidate biomarkers for discrimination of healthy subjects from LTBI and TB patients by employing microarray analysis and PCR analysis [6–7, 21, 24–25, 33, 35, 36]. In support of these reports, we indicate that a specific type of SOCS gene expresses differently between healthy individuals and TB subjects living in Taiwan. Expressions of SOCS-2, -3, -4, -5, -6, -7, and CIS-1 genes and their correlative relationship can help in understanding discrimination of healthy individuals from TB subjects, or discrimination of LTBI from active TB subjects. Although the different mRNA expression profiles of the eight SOCS molecules occur between TB and healthy subjects and possibly vary with the gender and age, SOCS-3 seems to be the best candidate among SOCS molecules for distinguishing male healthy individuals from LTBI subjects. As the SOCS family functions to act as negative regulators of cytokines [5], which can modulate immunological response of host to the *Mtb* infection [1,2,6,7], significant differences found in expressions of SOCS-2, -3, -4, -5, -6, -7, and CIS-1 genes among healthy, LTBI and active TB subjects may reflect the roles of the seven members of the SOCS family involved in immune regulation and cytokine signaling of human tuberculosis.
